# Analysis of deep grey nuclei susceptibility in early childhood: a quantitative susceptibility mapping and R2* study at 3 Tesla

**DOI:** 10.1007/s00234-021-02846-0

**Published:** 2021-11-17

**Authors:** Peter Raab, Stefan Ropele, Eva Bültmann, Rolf Salcher, Heinrich Lanfermann, Mike P. Wattjes

**Affiliations:** 1 Department of Diagnostic and Interventional Neuroradiology, Hannover Medical School, Carl-Neuberg-Str. 1, 30625 Hannover, Germany; 2 Clinical Department of Neurology, Medical University of Graz, Graz, Austria; 3 Clinic for Laryngology, Rhinology and Otology, Hannover Medical School, Hannover, Germany

**Keywords:** Brain, Iron, Aging, Child

## Abstract

**Purpose:**

Aging is the most significant determinant for brain iron accumulation in the deep grey matter. Data on brain iron evolution during brain maturation in early childhood are limited. The purpose of this study was to investigate age-related iron deposition in the deep grey matter in children using quantitative susceptibility (QSM) and R2* mapping.

**Methods:**

We evaluated brain MRI scans of 74 children (age 6–154 months, mean 40 months). A multi-echo gradient-echo sequence obtained at 3 Tesla was used for the QSM and R2* calculation. Susceptibility of the pallidum, head of caudate nucleus, and putamen was correlated with age and compared between sexes.

**Results:**

Susceptibility changes in all three nuclei correlated with age (correlation coefficients for QSM/R2*: globus pallidus 0.955/0.882, caudate nucleus 0.76/0.65, and putamen 0.643/0.611). During the first 2 years, the R2* values increased more rapidly than the QSM values, indicating a combined effect of iron deposition and myelination, followed by a likely dominating effect of iron deposition. There was no significant gender difference.

**Conclusion:**

QSM and R2* can monitor myelin maturation processes and iron accumulation in the deep grey nuclei of the brain in early life and may be a promising tool for the detection of deviations of this normal process. Susceptibility in the deep nuclei is almost similar early after birth and increases more quickly in the pallidum. The combined use of QSM and R2* analysis is beneficial.

## Introduction

Iron is a crucial trace element for the development and homeostasis of multiple organs including the central nervous system. Pathological iron deposition in the brain, particularly in deep grey matter structures, has been described in neuroinflammatory [1, 2] and neurodegenerative [3, 4] as well as metabolic diseases [5]. In addition, physiological iron deposition is related to normal aging [6].

During early life, the brain undergoes a process of maturation due to its incomplete development at birth. This maturation process includes the production of axonal myelin sheets within the white matter, which is dependent on the function of the oligodendrocytes. Oligodendrocytes represent the vast majority of iron-containing cells in the brain, found in perineuronal satellite positions as well as along white matter tracts [7]. Iron is not exclusively needed for myelin production with respect to cholesterol and lipid synthesis but serves also as an enzyme cofactor in the high metabolic activity of the brain [7]. The highest iron concentrations in the adult brain can be found within the deep grey nuclei [8] and the iron content changes during normal aging [9–11]. Iron deficiencies are the most frequent cause of deficiency-related diseases, leading possibly to hypomyelination, delayed cognitive or motor development in children [12–14]. While iron deposition does have a paramagnetic effect on tissue susceptibility, the effect of myelination is diamagnetic.

MRI using T2*- and susceptibility-weighted imaging are established tools for the noninvasive detection of iron-related signal intensity changes [11, 15, 16]. R2* mapping is another gradient-echo–derived measure that has been validated against iron content [17]. Quantitative susceptibility mapping (QSM) is the recently developed state-of-the-art technique for the quantitative and noninvasive estimation of iron within the tissue, allowing the determination of bulk magnetic susceptibility from gradient-echo phase images [18, 19]. Compared to R2* measurements, QSM does allow for the separation of paramagnetic and diamagnetic susceptibility effects based on different phase shifts.

The knowledge about the normal development of the magnetic susceptibility within the brain is of clinical importance given the intended use of QSM in routine diagnostic examinations.

Therefore, the aim of our study was the identification and analysis of age-related susceptibility changes of the deep grey nuclei in early childhood using the R2* evaluation and the QSM technique.

## Materials and methods

### Data set

All participants of this study were referred to our hospital due to hearing loss and underwent a diagnostic work-up during an evaluation for a possible cochlear implant therapy. Enrollment took place over a 24-month period, and every exam meeting the inclusion criteria was screened for inclusion of the study. Inclusion criteria were age < 15 years and completion of the routine MR examination during the cochlear implant evaluation procedure due to hearing impairment of unknown cause. Exclusion criteria were any brain abnormalities (e.g., signs of delayed or abnormal maturation, cerebral malformations, intracranial hemorrhage, CNS infection, parenchymal defects, or scarring), based on the medical history and the multi-sequence MR-protocol as evaluated by a neuroradiologist with more than 15 years of neuroradiological experience (PR). The parents of the children gave written informed consent to the scientific use of the data, and the need for an official IRB approval was waived due to the retrospective analysis nature of routine diagnostic imaging data.

### MR imaging

Brain MRI was performed under general anesthesia (except for one 13-year-old child) using a 3 Tesla whole-body MR system (Verio, Siemens, Erlangen, Germany) equipped with a 12-channel head coil. The MRI protocol consisted of an axial 2 dimensional (D)-fluid attenuated inversion recovery sequence (FLAIR, echo time [TE] 94 ms, repetition time [TR] 9000 ms, inversion time [TI] 2500 ms, slice thickness 4 mm), a 2D-triple echo T2-weighted sequence (TE 1–3: 8.7–70-131 ms, TR 5270 ms, slice thickness 4 mm), a diffusion-weighted sequence (b = 1000 smm^−2^, TE 94 ms, TR 8600 ms, slice thickness 4 mm), an ultrahigh resolution T2-weighted 3D-TSE sequence with slab selective variable excitation pulses (SPACE, TE 139 ms, TR 1000 ms, slice thickness 0.3 mm) centered at the inner ear structures, a T1-weighted sequence covering the petrous bone (TE 12 ms, TR 752 ms, slice thickness 2 mm), a coronal T2-weighted sequence (TE 92 ms, TR 5440 ms, slice thickness 4 mm), a sagittal T2-weighted sequence (TE 85 ms, TR 4000 ms, slice thickness 3 mm), and a 3D-T1–weighted magnetization-prepared rapid gradient-echo sequence (MPRAGE, TE 2.27 ms, TR 1800 ms, TI 900 ms, slice thickness 1 mm). For QSM and R2* calculations, a spoiled 3D-FLASH sequence with 8 equally spaced echoes was used (TE1-8: 3.6 ms, 9.51 ms, 15.43 ms, 21.34 ms, 27.26 ms, 33.17 ms, 39.09 ms, 45 ms; TR 55 ms; flip angle 15°, FOV 240^2^; slice thickness 2 mm; 80 slices; matrix 256^2^; parallel imaging factor 2).

### Image postprocessing and analysis

**Fig. 1:**
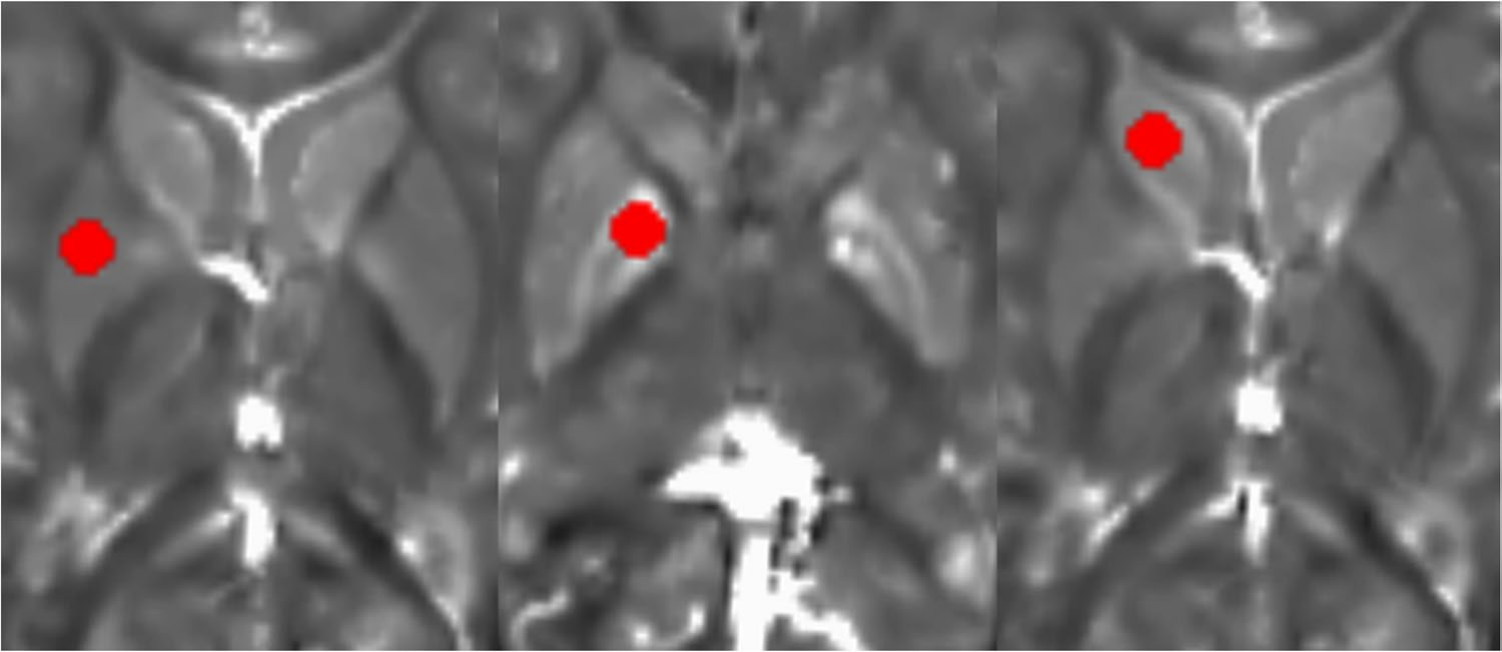
Typical ROI positions within the putamen (left), the globus pallidus (middle), and head of caudate nucleus (right)

The raw magnitude and phase images from the 3D-FLASH sequence were transferred to an offline workstation, and quantitative susceptibility maps were reconstructed using the morphology-enabled dipole inversion (MEDI + 0) – method with referencing of the parenchymal susceptibility values to the intraventricular cerebrospinal fluid susceptibility [20, 21]. This method is a stepwise procedure including a total field estimation by a nonlinear fit of the multi-echo data, local field computation by spatial field unwrapping, and background field removal using the projection onto dipole fields algorithm (PDF) followed by inversion of this result in order to get the susceptibility map. The MEDI + 0 – method uses magnitude image-based priors (edges) during the numerical inversion process as well as a regularization term leading to a uniform susceptibility distribution of CSF in order to enhance the QSM image quality (as it was also described in [5]). R2* calculation is based on the auto-regression on linear operations (ARLO) method [22], which is included in the same toolbox as the MEDI + 0 method, provided by the Cornell MRI Research Lab, Cornell University, USA (http://pre.weill.cornell.edu/mri/pages/qsm.html). Region of interest spheres with a radius of 3 mm were manually placed by two neuroradiologists individually (PR, MPW; with more than 15 years of neuroradiological experience) in the right putamen (PT), head of caudate nucleus (CN), and globus pallidus (GP) using MRIcro (Chris Rorden; www.mricro.com) and its multiplanar visualization function. The GP ROI was placed anteriorly and covered both the internal and external parts of this nucleus (Fig. 1). We analyzed exclusively the deep nuclei on the right in order to prevent possible effects of hemispheric differences with higher iron levels in left-sided deep nuclei, which were described in adults and were attributed possibly to motor lateralization [23]. Susceptibility and R2* values within the ROIs were extracted using FSL [24]; mean QSM and R2* values were calculated using the two ROI datasets for each anatomical location. The software “IBM SPSS statistics” (SPSS: IBM Corp. Released 2019, IBM SPSS Statistics for Windows, Version 26.0, Armonk, NY: IBM Corp.) was used to assess the correlation (calculation of Pearson correlation coefficient R using a linear regression analysis) between age and susceptibility as well as R2* values of the three individual nuclei for the whole patient group. The Mann–Whitney U test was used to test for susceptibility differences between female and male children within each region and a multiple regression analysis was used to assess a possible influence of sex on the correlation between age and susceptibility values for each region. The p value requested was adapted to p < 0.005 due to the number of tests.

## Results

**Fig. 2:**
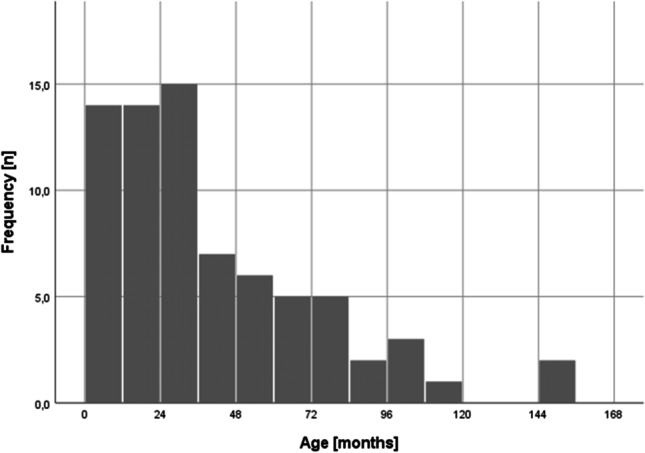
Histogram chart showing the age distribution of the included children

A total of 109 patients (age 6–154 months, mean 40 months) were screened. 23 datasets had to be excluded due to the exclusion criteria, and 12 datasets were excluded due to imaging artifacts (mainly distortions) resulting in 74 patients available for analysis (male/female: 45/29). 50/74 children were younger than 48 months. Figure 2 demonstrates the age distribution of our cohort using group sizes of 12 months. The included children below 36 months showed a 2:1 male predominance (Table 1). 57/74 children were diagnosed with a sensorineural hearing loss, whereas 4/74 of the children presented with conductive hearing loss. In 5/74 children the left side was affected, in 8/74 children the right, and in 48/74 children both sides were affected. 13/74 children had normal hearing capabilities.

**Table 1:** Susceptibility [in ppb] and R2* [in Hz] of the three deep grey nuclei for different age groups (both sexes grouped together as well as separated for females and males)

			QSM			R2*		
Age [months]	Gender	Number of children (total 74)	Head of caudate (mean (SD))	Globus pallidum (mean (SD))	Putamen (mean (SD))	Head of caudate (mean (SD))	Globus pallidum (mean (SD))	Putamen (mean (SD))
**0–12**	Total	14	− 8.2 (9.3)	− 17.4 (5.7)	− 29.4 (4.9)	10.8 (0.5)	10.7 (0.7)	11.3 (0.5)
	Female	5	− 9.2 (9.9)	− 16.1 (6.3)	− 26.0 (4.5)	10.7 (0.5)	10.5 (0.6)	11.1 (0.7)
	Male	9	− 7.7 (9.5)	− 18.1 (5.7)	− 26.9 (5.4)	10.8 (0.6)	10.8 (0.8)	11.4 (0.5)
**13–24**	Total	14	− 10.1 (7.7)	− 15.0 (6.8)	− 26.18 (6.7)	12.1 (0.7)	13.2 (1.7)	12.7 (1.0)
	Female	4	− 7 (9.7)	− 18.6 (2.9)	− 27.9 (6.4)	11.7 (0.7)	12.1 (0.7)	12 (0.5)
	Male	10	− 11.4 (7)	− 13.6 (7.5)	− 25.3 (7)	12.2 (0.7)	13.6 (1.8)	13 (1)
**25–36**	Total	15	− 6.2 (8)	− 6.7 (9.6)	− 26 (9.6)	13 (2.3)	15.3 (2.3)	13.7 (1.9)
	Female	5	− 5.4 (9.7)	− 7.8 (7.8)	− 27.9 (5.3)	12.8 (0.9)	15 (1.6)	13.4 (0.9)
	Male	10	− 6.6 (7.6)	− 6.1 (10.8)	− 25 (10.5)	13.1 (2.8)	15.4 (2.6)	13.8 (2.2)
**37–48**	Total	7	− 3.7 (5.9)	13.3 (9.9)	− 22.6 (9.2)	13.1 (0.3)	16.2 (0.8)	14.2 (0.5)
	Female	3	0.1 (4.8)	19.9 (10)	− 17.4 (8.5)	13.2 (0.3)	16.6 (0.6)	14.1 (0.2)
	Male	4	− 6.4 (5.6)	8.4 (7.3)	− 26.5 (6.9)	13 (0.4)	15.9 (0.8)	14.2 (0.7)
**49–60**	Total	6	1.8 (4.9)	20.7 (6.7)	− 24.6 (4.3)	13.4 (0.7)	18.4 (0.4)	14.4 (0.6)
	Female	5	3.1 (4.2)	19.6 (6.9)	− 25.2 (4.6)	13.3 (0.7)	18.3 (0.3)	14.4 (0.7)
	Male	1	− 4.6	26.4	− 21.5	13.8	19	14.5
**61–72**	Total	5	9.1 (6.3)	62.7 (14.5)	− 8.2 (6.6)	14.6 (0.5)	21.8 (1.7)	15.4 (0.5)
	Female	3	11.2 (1.7)	65.6 (20.5)	− 3.7 (6.8)	14.8 (0.4)	22.3 (2.7)	15.7 (0.6)
	Male	2	7.7 (8.3)	60.8 (14)	− 11.2 (5.5)	14.4 (0.6)	21.5 (1.1)	15.2 (0.4)
**73–84**	Total	5	14.7 (14.1)	68.8 (14)	− 8 (10.3)	14.7 (0.9)	22 (1.6)	15.2 (0.6)
	Female	3	15.5 (19.8)	69.3 (17.9)	− 6.2 (11.9)	14.8 (1.2)	22.6 (1.8)	15.4 (0.8)
	Male	2	13.7 (2.2)	68 (11.9)	− 10.7 (10.7)	14.5 (0.3)	21.3 (1.4)	15 (0.1)
**85–96**	Total	2	18.8 (2.6)	105.8 (18.9)	− 11.1 (6.1)	15.3 (0.6)	24.7 (0.8)	15.6 (0.5)
	Female	0						
	Male	2	18.8 (2.6)	105.8 (18.9)	− 11.1 (6.1)	15.3 (0.6)	24.7 (0.8)	15.6 (0.5)
**97–108**	Total	3	20.4 (3.6)	85.7 (10.2)	− 6.5 (3.6)	13.8 (2.9)	20.5 (8.6)	14.1 (2.8)
	Female	1	23.5	84.6	− 10.4	16.1	26	16.4
	Male	2	18.9 (3.4)	86.3 (14.4)	− 4.5 (1.6)	12.7 (3)	17.8 (10)	12.9 (2.7)
** > 109**	Total	3	22.9 (16.5)	97.6 (39)	− 4.4 (25.8)	16.2 (1)	28.5 (2.8)	15.6 (2.2)
	Female	1	36.6	142	25	17.2	31.3	13.1
	Male	2	16 (16.2)	75.4 (11.8)	− 19.1 (6)	15.7 (0.7)	27 (2)	16.8 (0.7)

**Fig. 3:**
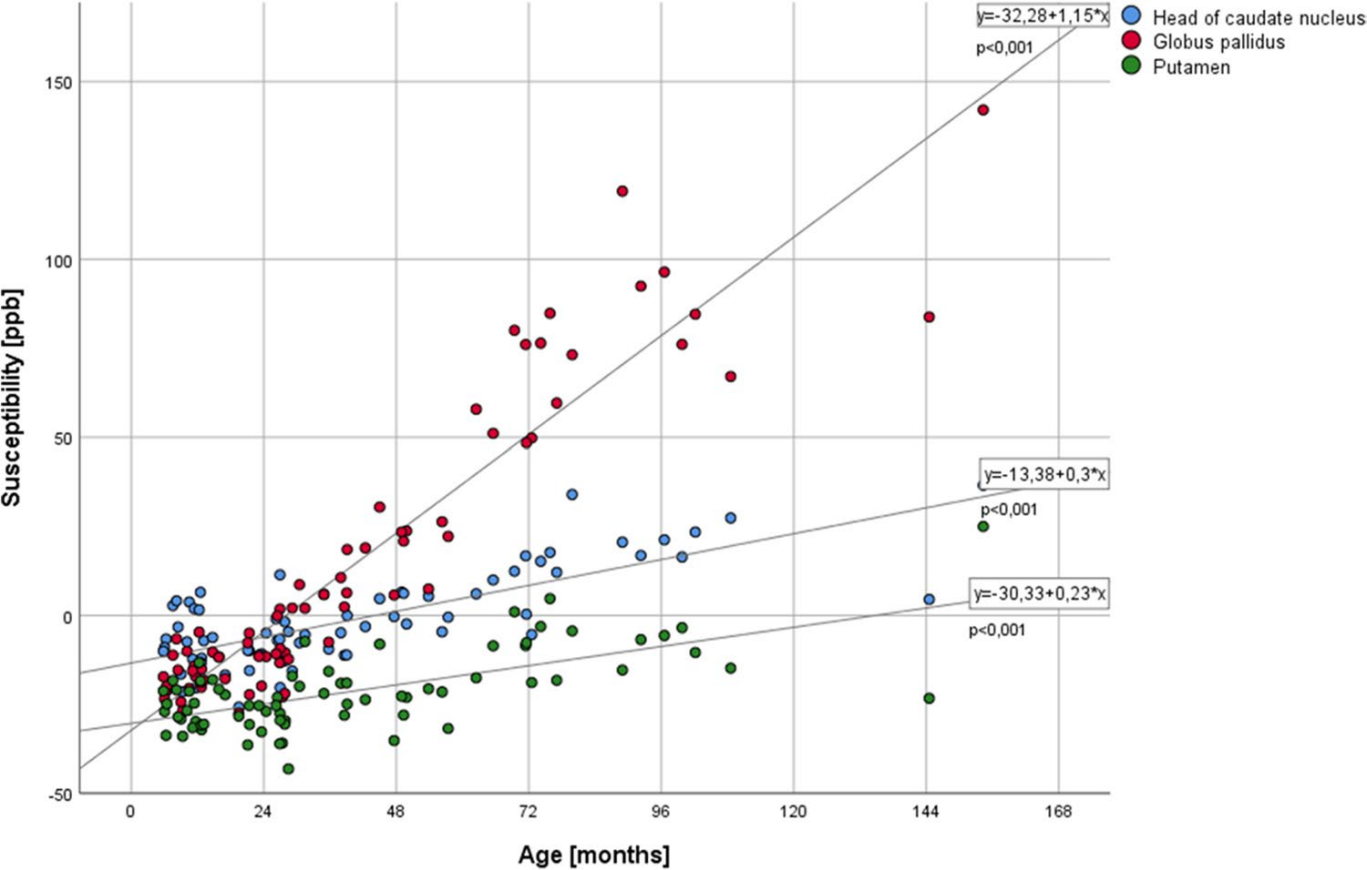
Diagram showing the change of the QSM data in relation to age. The equations relate to the linear fit of the correlation

In very young children (grouped ages 0–12 months, *n* = 14), the quantitative susceptibility was very similar for the three deep grey matter structures with the highest susceptibility in the CN (mean ± SD: -8.2 ± 9.43 [all susceptibilities in ppb]) and the lowest in PT vs. GP (-29.4 ± 4.9 vs. -17.4 ± 5.7, respectively). The fastest susceptibility increase occurred within the GP, leading to a mean susceptibility value of 97.6 ± 39 in the 3 children older than 9 years (see Table 1 and Fig. 3). This is also represented by the highest slope of the correlation between age and susceptibility for GP (*y* = -32.28 + 1.15*x) as compared to the other two nuclei (CN: *y* = -13.38 + 0.3*x; PT: *y* = -30.33 + 0.23*x) (Fig. 3).

**Fig. 4:**
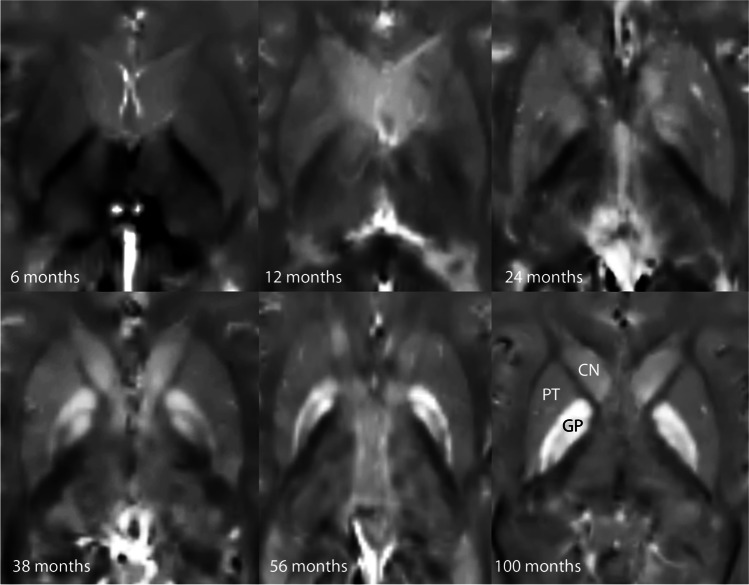
QSM images demonstrating the susceptibility changes with increasing age within the three deep grey structures/nuclei/regions (nuclei: globus pallidus (GP), putamen (PT), and head of caudate nucleus (CN))

Among the three nuclei, the Pearson correlation coefficient R between susceptibility and age was the highest for GP (*R* = 0.955 with p < 0.001, *R*^2^ 0.913, *R*^2^ corrected 0.911), followed by CN (0.76, *p* < 0.001, *R*^2^ 0.578, *R*^2^ corrected 0.569), and Putamen (0.643, *p* < 0.001, *R*^2^ 0.413, *R*^2^ corrected 0.401). At the age of 9 to 10 years, the relative susceptibility of the GP is higher compared to PT and CN (see Fig. 4 demonstrating the QSM signal characteristics for different ages).

**Fig. 5:**
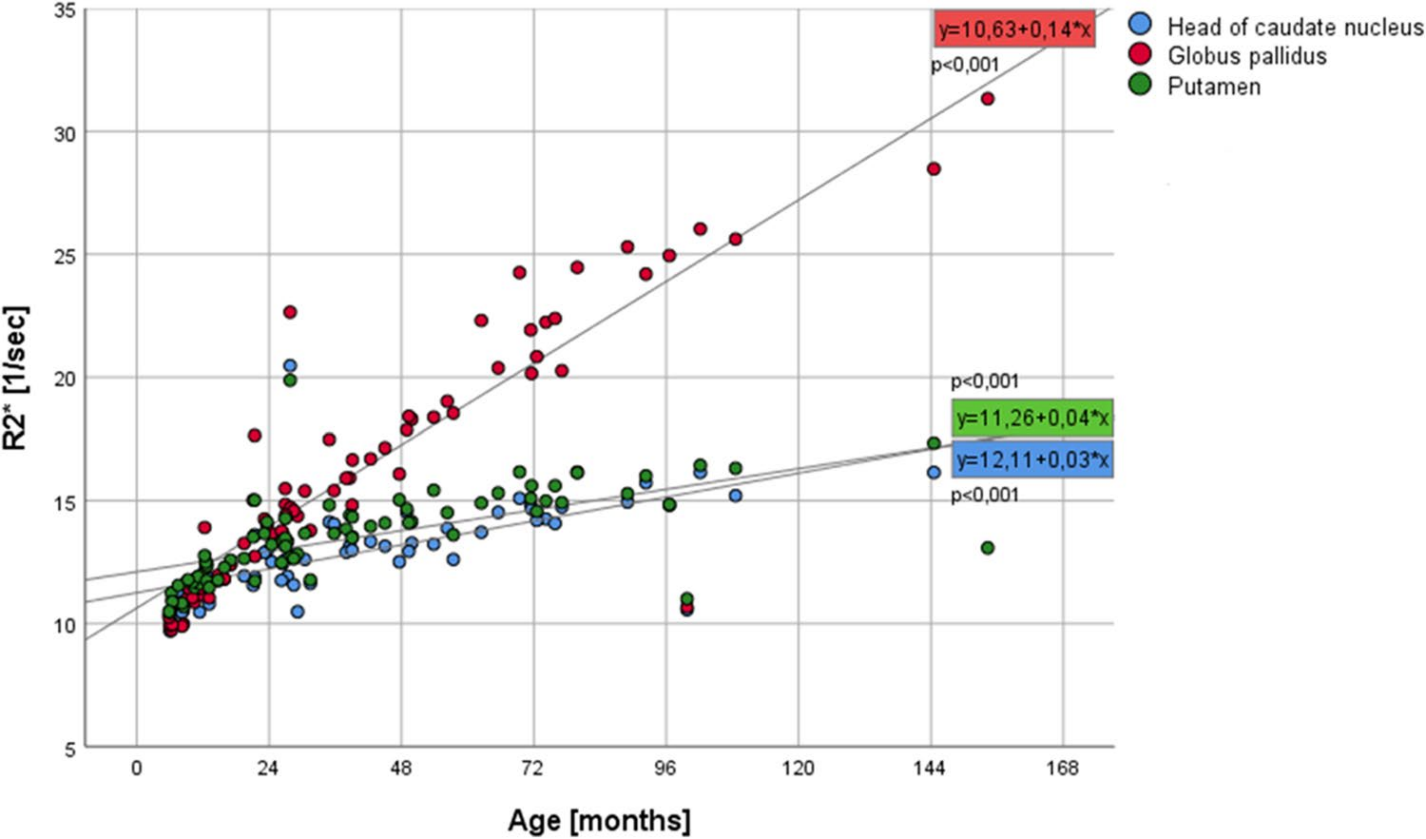
Diagram showing the change of the R2* values in relation to age. The equations relate to the linear fit of the correlation. Some outliers are included in the diagram

**Fig. 6:**
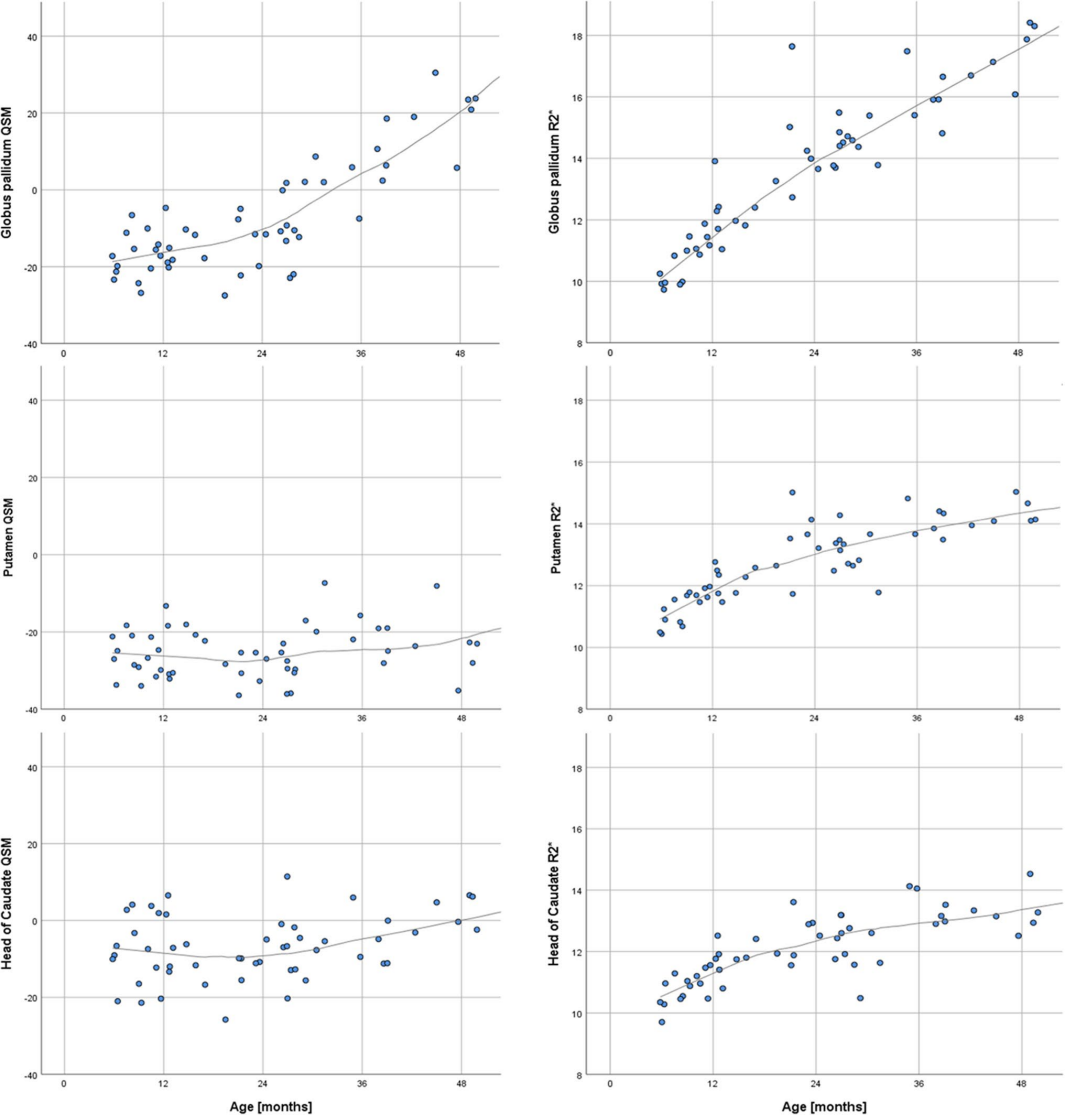
Diagram showing the change of QSM and R2* values during the first 4 years of life within the ROI’s of GP, PT, and CN (Loess-curve fitting by SPSS). For GP (top row), R2* values do increase more rapidly during the first 2 years compared to the almost unchanged QSM values, suggesting a combined change of diamagnetic and paramagnetic effects in the tissue. For PT (middle row), R2* values also do increase more rapidly during the first two years compared to the QSM values, also suggesting a combined change of diamagnetic and paramagnetic effects in the tissue. QSM value increase occurs later compared to the GP. For CN (bottom row), R2* values again do increase more rapidly during the first 2 years compared to the QSM values. QSM values start to increase after 24 months of age, comparable to PT

R2* values of the three grey nuclei are about the same in the youngest age group (CN 10.8 ± 0.5; GP 10.7 ± 0.7; PT 11.3 ± 0.5). The biggest increase of R2* does occur in the GP (Fig. 5) similar to the susceptibility values, although the change over time is much slower. R2* values do correlate highly significant (*p* < 0.001) with age (Pearson’s correlation coefficients: CN 0.695; GP 0.882; PT 0.611). For the age group 0–24 months, the CN R2* values change with a higher Pearson’s coefficient with respect to age (for CN Pearson’s *R* = 0.817, *p* < 0.001; for PT as well as GP *R* = 0.842, *p* < 0.001, respectively). The R2* values increase faster during this early time period and slower for later time points. The QSM values, on the other hand, increase slower or even decrease for this early time period but increase faster for later time points (Fig. 6).

A multiple regression analysis with sex as a covariate revealed that there was no significant effect of sex on the susceptibility or R2* values of the deep grey matter nuclei. Using the Mann–Whitney U test, there was also no significant difference between the susceptibilities or R2* values of the three deep nuclei between the female and male children at any age interval (0.4 < *p* < 0.8; susceptibility and R2* values are shown in Table 1).

## Discussion

Development and function of the brain depend on the presence of iron, which is a trace element needed to be taken up with nutrition [12]. Uptake into the brain is highly regulated [25] and region specific; most of the iron is found in oligodendrocytes close to neurons and within white matter tracts [7]. Iron is needed for myelin production, and therefore it is important during the process of brain maturation. R2* values in deep grey nuclei have been shown to increase due to iron deposition with aging in young children [26], but also myelination is leading to increasing R2* values and thereby possibly to an overestimation of the existing iron content [27, 28]. QSM is sensitive to diamagnetic and paramagnetic changes within the tissue due to the inclusion of phase change information and thereby can indicate changes of iron and myelin content, although this method is not specific for both of them. It has been demonstrated that myelin does cause diamagnetic rather than paramagnetic effects in QSM imaging [29], thereby allowing to attribute potential QSM signal increases to iron, since ongoing myelination should decrease the QSM signal. In the case of mixed effects by parallel myelination and iron deposition, QSM might not change overall. Therefore, the combination of R2* and QSM analyses might be favorable in order to detect iron and myelination-induced susceptibility changes also during early brain development in particular in deep grey matter, as it was suggested for adult aging by Betts et al. [27].

In this study, we investigated the association between the age of very young children (ages 0 to 9 years) and the bulk susceptibility of deep grey nuclei by using the recently introduced MEDI + 0 – QSM reconstruction algorithm as well as R2* calculation by using the identical MR dataset. The MEDI + 0 reconstruction method, which includes an automatic cerebrospinal fluid (CSF) zero referencing, has been shown to provide a more accurate quantification in the region of deep grey nuclei by reducing artifacts close to the ventricular wall compared to the prior MEDI method [21]. Using CSF as a reference instead of white matter omits the confounding effect of ongoing myelination with its increasing diamagnetic susceptibility effect on the analysis results.

The relationship between brain iron content and aging has been the aim of several studies, ranging from the susceptibility in elderly to adolescents and also a few studies in children [8, 10, 11, 27, 30–35]. If QSM was used in these studies, the mathematical methods of the QSM calculation were different. Most of these studies lack information on very young children, except for Ning et al. [35]. Betts et al. [27] concluded in their study of aging adults that although QSM can potentially advance the knowledge of iron dysregulation in aging, the combination of QSM and R2* analysis could provide a more complete picture of iron and myelination changes.

We found very similar susceptibility values within the three tested deep nuclei in the youngest children of our cross-sectional cohort, which increased age-related more rapidly in the globus pallidus compared to the head of the caudate nucleus and the putamen leading to a recognizable difference at the age range of 50–100 months. Although our method is different to Ning et al., we found almost the same slope of the correlation between age and susceptibility in the globus pallidus. The increase of the QSM values of the putamen and caudate nucleus developed with almost the same slope, differing only by a slightly higher level of paramagnetic susceptibility in the caudate than in the putamen. Interestingly, the QSM values of the globus pallidus and putamen did start at a more negative susceptibility compared to the head of the caudate nucleus in our study, which is also in accordance with data published by Ning et al. [35]. The R2* values in our study showed similar changes with increasing age compared to data found by Ning et al. [26], but the slope of the R2* increase overall is smaller compared to the QSM data. This is completely different when looking at the age group 0–24 months. R2* values do increase more rapidly than the almost unchanged QSM values during this time span, indicating a mixed effect of increasing diamagnetic and paramagnetic susceptibility changes caused by a parallel development of myelination and iron deposition. After 2 years, the myelination process has usually finished and the ongoing iron deposition is indicated by the combined increase of QSM and R2* values.

Since there is virtually no iron accumulated in the tissue at birth as well as the nonmyelinated tissue situation, the tissue susceptibility in the youngest children might be more influenced by the cellular composition of the tissue, and maybe lower numbers of oligodendrocytes within the caudate nucleus compared to the other two nuclei are responsible for the low CN-QSM values since in adults this histologic difference has been described by Blinkov and Glezer [36]. Several studies reported sex related differences in the susceptibility of deep grey matter nuclei in adults as well [16, 30, 37, 38], showing lower susceptibility effects in women. We found no significant gender effect on the susceptibility of the children in our study, indicating that possible differences found later in life [16, 37] might be acquired possibly depending on previously discussed hormonal differences or menstruation and that a genetic cause of the differences between sexes later during life might be less likely. The apparent difference of susceptibility values between male and female in the oldest group (> 109 months, Table 1) cannot be accounted for due to the small number of individuals.

Although several studies have concentrated on the age-related iron storage in the brain, the exact mechanism is not yet fully understood. The iron in the brain tissue located in non-erythroid cells is mainly stored within ferritin [4], which is a protein shell allowing the storage of iron in a water soluble, nontoxic, and bioavailable form. Ferritin can be made of two polypeptide chains (heavy = H; light = L), which serve different functions [4] within the cells. The L-form is attributed to storage and the H-form is attributed to stress-related Fe2 + -/Fe3 + -oxidation. The expression of the two subtypes can differ between cell types of the brain [4, 39] with almost equal amounts of H- and L-types within oligodendrocytes and more H-type within neurons. An increase of the amount of these subtypes within the cells could be demonstrated during aging, especially for the L-type in the globus pallidus [40, 41]. This may contribute to the different iron content in the deep grey matter nuclei during brain maturation, possibly paralleling the development of cognitive functions and motor skills of the children [35, 42].

Our study has some limitations. First of all, most of the included children presented with either sensorineural or conductive hearing impairment and were therefore not entirely healthy, but depending on the screening results including the evaluation of the multisequence brain MRI and the extensive otologic diagnostic work-up, we reliably excluded confounding effects like delayed cerebral maturation, cerebral malformations, or other cerebral causes. Therefore, the hearing impairment of the included children can be assumed to be attributed to the middle ear, the cochlea, or the cochlear nerve itself, and this should not have a confounding effect on our analysis. Except for one 13-year-old female, all study participants were scanned under general anesthesia, which was a requirement for the cochlea implant evaluation process including invasive hearing tests as well as possible surgical ENT procedures. A hyperoxic effect of a susceptibility decrease by 10% in rat brains when compared to normoxia has been described [43]. Therefore, our susceptibility measurements could artificially be slightly to low, which might at least in part be accounted for by the MEDI + 0 – referencing method. Since the susceptibility measurement of the three nuclei is made intraindividually, the affirmative finding of the more rapid susceptibility increases of the globus pallidus with age compared to the other two nuclei is independent from a possible hyperoxia during the MRI. Manual ROI placement is time-consuming and prone to placement errors, and an automatic segmentation method is desirable, but an automatic segmentation of deep nuclei is difficult especially in cases of low susceptibility and to our knowledge no QSM-based atlases are available. Although established brain parcellation methods like FreeSurfer (https://surfer.nmr.mgh.harvard.edu/) and ANT’s (by University of Pennsylvania; http://stnava.github.io/ANTs/) offer T1w-based templates for children with young ages, these segmentation results would have to be corrected for distortion differences between T1w-3D images and QSM images leading to possible errors. We therefore used two sets of ROI’s placed by two individuals and used the mean values for analysis. Another limitation is the cross-sectional study design, which is based on the inability to perform a longitudinal study on children at this age. Future studies should aim at characterizing different age windows like 0–2 years, 2–6 years, and 6–12 years.

In conclusion, the knowledge of susceptibility changes within the brain and the separation of iron concentration and myelination related changes with age is an important baseline for the understanding of neurodegeneration and related diseases like neurodegeneration with brain iron accumulation (NBIA) in children [44] or Alzheimer’s disease and Parkinson’s disease in adults and elderly [45–47]. Our study can add to the knowledge about iron-related susceptibility changes of deep grey nuclei during brain maturation early in life and it supports recent data. The susceptibility of the globus pallidus starts at a slightly lower level compared to the head of the caudate nucleus and increases more rapidly than the susceptibility of the putamen and head of caudate with age and brain maturation. During the first 2 years of life, susceptibility changes in the deep nuclei represent a combination of parallel diamagnetic and paramagnetic effects, e.g., caused by iron deposition as well as myelination. This research provides the basic knowledge for the evaluation of diseases with either iron deficiency or iron overload, especially in children at a very young age, and indicates a benefit of the combination of QSM and R2* analysis for these questions.

## Data Availability

Does not apply.

## Abbreviations

2D: Two dimensional
3D: Three dimensional
CN: Head of caudate nucleus
CNS: Central nervous system
ENT: Ear-nose-throat
FLASH: Fast low-angle shot
FOV: Field of view
FSL: Functional MRI of the Brain Software Library
GP: Globus pallidus
H: Heavy
IRB: Institutional review board
L: Light
MEDI: Morphology enabled dipole inversion
MPRAGE: Magnetization-prepared rapid gradient-echo
MR: Magnetic resonance
MRI: Magnetic resonance imaging
NBIA: Neurodegeneration with brain iron accumulation
ppb: Parts per billion
PT: Putamen
QSM: Quantitative susceptibility mapping
ROI: Region of interest
SD: Standard deviation
TE: Echo time
TI: Inversion time
TR: Repetition time
TSE: Turbo spin echo
vs.: Versus

## References

[1] Ropele S, Kilsdonk ID, Wattjes MP, Langkammer C, de Graaf WL, Frederiksen JL, Larsson HB, Yiannakas M, Wheeler-Kingshott CA, Enzinger C, Khalil M, Rocca MA, Sprenger T, Amann M, Kappos L, Filippi M, Rovira A, Ciccarelli O, Barkhof F, Fazekas F (2014). Determinants of iron accumulation in deep grey matter of multiple sclerosis patients. Mult Scler.

[2] Schweser F, Hagemeier J, Dwyer MG, Bergsland N, Hametner S, Weinstock-Guttman B, Zivadinov R (2021). Decreasing brain iron in multiple sclerosis: the difference between concentration and content in iron MRI. Hum Brain Mapp.

[3] Thomas M, Jankovic J (2004). Neurodegenerative disease and iron storage in the brain. Curr Opin Neurol.

[4] Zecca L, Youdim MB, Riederer P, Connor JR, Crichton RR (2004). Iron, brain ageing and neurodegenerative disorders. Nat Rev Neurosci.

[5] Li J, Zhang Q, Zhang N, Guo L (2020). Increased brain iron deposition in the putamen in patients with type 2 diabetes mellitus detected by quantitative susceptibility mapping. J Diabetes Res.

[6] Bartzokis G, Beckson M, Hance DB, Marx P, Foster JA, Marder SR (1997). MR evaluation of age-related increase of brain iron in young adult and older normal males. Magn Reson Imaging.

[7] Connor JR, Menzies SL (1996). Relationship of iron to oligodendrocytes and myelination. Glia.

[8] Bilgic B, Pfefferbaum A, Rohlfing T, Sullivan EV, Adalsteinsson E (2012). MRI estimates of brain iron concentration in normal aging using quantitative susceptibility mapping. Neuroimage.

[9] Hallgren B, Sourander P (1958). The effect of age on the non-haemin iron in the human brain. J Neurochem.

[10] Liu M, Liu S, Ghassaban K, Zheng W, Dicicco D, Miao Y, Habib C, Jazmati T, Haacke EM (2016). Assessing global and regional iron content in deep gray matter as a function of age using susceptibility mapping. J Magn Reson Imaging.

[11] Hect JL, Daugherty AM, Hermez KM, Thomason ME (2018). Developmental variation in regional brain iron and its relation to cognitive functions in childhood. Dev Cogn Neurosci.

[12] Beard JL, Connor JR (2003). Iron status and neural functioning. Annu Rev Nutr.

[13] Wolf NI, Ffrench-Constant C, van der Knaap MS (2021). Hypomyelinating leukodystrophies - unravelling myelin biology. Nat Rev Neurol.

[14] Tang S, Xu Y, Liu X, Chen Z, Zhou Y, Nie L, He L (2021). Quantitative susceptibility mapping shows lower brain iron content in children with autism. Eur Radiol.

[15] Haacke EM, Cheng NY, House MJ, Liu Q, Neelavalli J, Ogg RJ, Khan A, Ayaz M, Kirsch W, Obenaus A (2005). Imaging iron stores in the brain using magnetic resonance imaging. Magn Reson Imaging.

[16] Larsen B, Bourque J, Moore TM, Adebimpe A, Calkins ME, Elliott MA, Gur RC, Gur RE, Moberg PJ, Roalf DR, Ruparel K, Turetsky BI, Vandekar SN, Wolf DH, Shinohara RT, Satterthwaite TD (2020). Longitudinal development of brain iron is linked to cognition in youth. J Neurosci.

[17] Langkammer C, Krebs N, Goessler W, Scheurer E, Ebner F, Yen K, Fazekas F, Ropele S (2010). Quantitative MR imaging of brain iron: a postmortem validation study. Radiology.

[18] Langkammer C, Schweser F, Krebs N, Deistung A, Goessler W, Scheurer E, Sommer K, Reishofer G, Yen K, Fazekas F, Ropele S, Reichenbach JR (2012). Quantitative susceptibility mapping (QSM) as a means to measure brain iron? A post mortem validation study. Neuroimage.

[19] Liu C, Li W, Tong KA, Yeom KW, Kuzminski S (2015). Susceptibility-weighted imaging and quantitative susceptibility mapping in the brain. J Magn Reson Imaging.

[20] Liu J, Liu T, de Rochefort L, Ledoux J, Khalidov I, Chen W, Tsiouris AJ, Wisnieff C, Spincemaille P, Prince MR, Wang Y (2012). Morphology enabled dipole inversion for quantitative susceptibility mapping using structural consistency between the magnitude image and the susceptibility map. Neuroimage.

[21] Liu Z, Spincemaille P, Yao Y, Zhang Y, Wang Y (2018). MEDI+0: Morphology enabled dipole inversion with automatic uniform cerebrospinal fluid zero reference for quantitative susceptibility mapping. Magn Reson Med.

[22] Pei M, Nguyen TD, Thimmappa ND, Salustri C, Dong F, Cooper MA, Li J, Prince MR, Wang Y (2015). Algorithm for fast monoexponential fitting based on Auto-Regression on Linear Operations (ARLO) of data. Magn Reson Med.

[23] Xu X, Wang Q, Zhang M (2008). Age, gender, and hemispheric differences in iron deposition in the human brain: an in vivo MRI study. Neuroimage.

[24] Jenkinson M, Beckmann CF, Behrens TE, Woolrich MW, Smith SM (2012). Fsl Neuroimage.

[25] Andrews NC, Schmidt PJ (2007). Iron homeostasis. Annu Rev Physiol.

[26] Ning N, Zhang L, Gao J, Zhang Y, Ren Z, Niu G, Dai Y, Wu EX, Guo Y, Yang J (2014). Assessment of iron deposition and white matter maturation in infant brains by using enhanced T2 star weighted angiography (ESWAN): R2* versus phase values. PLoS One.

[27] Betts MJ, Acosta-Cabronero J, Cardenas-Blanco A, Nestor PJ, Duzel E (2016). High-resolution characterisation of the aging brain using simultaneous quantitative susceptibility mapping (QSM) and R2* measurements at 7T. Neuroimage.

[28] Deistung A, Schafer A, Schweser F, Biedermann U, Turner R, Reichenbach JR (2013). Toward in vivo histology: a comparison of quantitative susceptibility mapping (QSM) with magnitude-, phase-, and R2*-imaging at ultra-high magnetic field strength. Neuroimage.

[29] Hametner S, Endmayr V, Deistung A, Palmrich P, Prihoda M, Haimburger E, Menard C, Feng X, Haider T, Leisser M, Kock U, Kaider A, Hoftberger R, Robinson S, Reichenbach JR, Lassmann H, Traxler H, Trattnig S, Grabner G (2018). The influence of brain iron and myelin on magnetic susceptibility and effective transverse relaxation - a biochemical and histological validation study. Neuroimage.

[30] Persson N, Wu J, Zhang Q, Liu T, Shen J, Bao R, Ni M, Liu T, Wang Y, Spincemaille P (2015). Age and sex related differences in subcortical brain iron concentrations among healthy adults. Neuroimage.

[31] Acosta-Cabronero J, Betts MJ, Cardenas-Blanco A, Yang S, Nestor PJ (2016). In vivo MRI mapping of brain iron deposition across the adult lifespan. J Neurosci.

[32] Darki F, Nemmi F, Moller A, Sitnikov R, Klingberg T (2016). Quantitative susceptibility mapping of striatum in children and adults, and its association with working memory performance. Neuroimage.

[33] Zhang Y, Wei H, Cronin MJ, He N, Yan F, Liu C (2018). Longitudinal atlas for normative human brain development and aging over the lifespan using quantitative susceptibility mapping. Neuroimage.

[34] Zhang Y, Wei H, Cronin MJ, He N, Yan F, Liu C (2018). Longitudinal data for magnetic susceptibility of normative human brain development and aging over the lifespan. Data Brief.

[35] Ning N, Liu C, Wu P, Hu Y, Zhang W, Zhang L, Li M, Gho SM, Kim DH, Guo H, Yang J, Jin C (2019). Spatiotemporal variations of magnetic susceptibility in the deep gray matter nuclei from 1 month to 6 years: a quantitative susceptibility mapping study. J Magn Reson Imaging.

[36] Blinkov SM, Glezer II (1968). The human brain in figures and tables. A quantitative handbook.

[37] Gong NJ, Wong CS, Hui ES, Chan CC, Leung LM (2015). Hemisphere, gender and age-related effects on iron deposition in deep gray matter revealed by quantitative susceptibility mapping. NMR Biomed.

[38] Darnai G, Nagy SA, Horvath R, Acs P, Perlaki G, Orsi G, Kovacs N, Altbacker A, Plozer E, Tenyi D, Weintraut R, Schwarcz A, John F, Varga E, Bereczkei T, Clemens Z, Komoly S, Janszky J (2017). Iron concentration in deep gray matter structures is associated with worse visual memory performance in healthy young adults. J Alzheimers Dis.

[39] Connor JR, Boeshore KL, Benkovic SA, Menzies SL (1994). Isoforms of ferritin have a specific cellular distribution in the brain. J Neurosci Res.

[40] Zecca L, Gallorini M, Schunemann V, Trautwein AX, Gerlach M, Riederer P, Vezzoni P, Tampellini D (2001). Iron, neuromelanin and ferritin content in the substantia nigra of normal subjects at different ages: consequences for iron storage and neurodegenerative processes. J Neurochem.

[41] Connor JR, Snyder BS, Arosio P, Loeffler DA, LeWitt P (1995). A quantitative analysis of isoferritins in select regions of aged, parkinsonian, and Alzheimer's diseased brains. J Neurochem.

[42] Leisman G, Braun-Benjamin O, Melillo R (2014). Cognitive-motor interactions of the basal ganglia in development. Front Syst Neurosci.

[43] Hsieh MC, Kuo LW, Huang YA, Chen JH (2017). Investigating hyperoxic effects in the rat brain using quantitative susceptibility mapping based on MRI phase. Magn Reson Med.

[44] Gregory A, Hayflick SJ (2005). Neurodegeneration with brain iron accumulation. Folia Neuropathol.

[45] Schenck JF, Zimmerman EA (2004). High-field magnetic resonance imaging of brain iron: birth of a biomarker?. NMR Biomed.

[46] Schroder N, Figueiredo LS, de Lima MN (2013). Role of brain iron accumulation in cognitive dysfunction: evidence from animal models and human studies. J Alzheimers Dis.

[47] Zhu WZ, Zhong WD, Wang W, Zhan CJ, Wang CY, Qi JP, Wang JZ, Lei T (2009). Quantitative MR phase-corrected imaging to investigate increased brain iron deposition of patients with Alzheimer disease. Radiology.

